# Combined Subthalamic and Nigral Stimulation Modulates Temporal Gait Coordination and Cortical Gait-Network Activity in Parkinson’s Disease

**DOI:** 10.3389/fnhum.2022.812954

**Published:** 2022-02-28

**Authors:** Jonas R. Wagner, Miriam Schaper, Wolfgang Hamel, Manfred Westphal, Christian Gerloff, Andreas K. Engel, Christian K. E. Moll, Alessandro Gulberti, Monika Pötter-Nerger

**Affiliations:** ^1^Department of Neurology, University Medical Center Hamburg-Eppendorf, Hamburg, Germany; ^2^Department of Neurosurgery, University Medical Center Hamburg-Eppendorf, Hamburg, Germany; ^3^Department of Neurophysiology and Pathophysiology, University Medical Center Hamburg-Eppendorf, Hamburg, Germany

**Keywords:** freezing of gait (FOG), Parkinson’s disease, deep brain stimulation, subthalamic nucleus, substantia nigra, electroencephalography, stepping in place, beta oscillations

## Abstract

**Background:**

Freezing of gait (FoG) is a disabling burden for Parkinson’s disease (PD) patients with poor response to conventional therapies. Combined deep brain stimulation of the subthalamic nucleus and substantia nigra (STN+SN DBS) moved into focus as a potential therapeutic option to treat the parkinsonian gait disorder and refractory FoG. The mechanisms of action of DBS within the cortical-subcortical-basal ganglia network on gait, particularly at the cortical level, remain unclear.

**Methods:**

Twelve patients with idiopathic PD and chronically-implanted DBS electrodes were assessed on their regular dopaminergic medication in a standardized stepping in place paradigm. Patients executed the task with DBS switched off (STIM OFF), conventional STN DBS and combined STN+SN DBS and were compared to healthy matched controls. Simultaneous high-density EEG and kinematic measurements were recorded during resting-state, effective stepping, and freezing episodes.

**Results:**

Clinically, STN+SN DBS was superior to conventional STN DBS in improving temporal stepping variability of the more affected leg. During resting-state and effective stepping, the cortical activity of PD patients in STIM OFF was characterized by excessive over-synchronization in the theta (4–8 Hz), alpha (9–13 Hz), and high-beta (21–30 Hz) band compared to healthy controls. Both active DBS settings similarly decreased resting-state alpha power and reduced pathologically enhanced high-beta activity during resting-state and effective stepping compared to STIM OFF. Freezing episodes during STN DBS and STN+SN DBS showed spectrally and spatially distinct cortical activity patterns when compared to effective stepping. During STN DBS, FoG was associated with an increase in cortical alpha and low-beta activity over central cortical areas, while with STN+SN DBS, an increase in high-beta was prominent over more frontal areas.

**Conclusions:**

STN+SN DBS improved temporal aspects of parkinsonian gait impairment compared to conventional STN DBS and differentially affected cortical oscillatory patterns during regular locomotion and freezing suggesting a potential modulatory effect on dysfunctional cortical-subcortical communication in PD.

## Introduction

Freezing of gait (FoG) is a sudden and episodic inability to produce effective forward stepping movements and is most commonly experienced in Parkinson’s disease (PD). FoG typically occurs during gait initiation, turning, and gait adjustments (Nutt et al., 2011). Different freezing phenotypes exist including high-frequency leg shuffling, trembling in place, and akinetic freezing (Schaafsma et al., 2003). Due to its unpredictable nature, FoG increases the risk of falls and hospitalization (Latt et al., 2009) and causes a substantial reduction in quality of life (Moore et al., 2007).

Normal gait function is enabled by effective communication within a large-scale functional system of cortical, subcortical, and spinal hubs (Snijders et al., 2016; Takakusaki, 2017). During steady-state walking, automatic gait control is achieved by downstream projections from the mesencephalic locomotor area (MLR) and pedunculopontine nucleus (PPN) to spinal central pattern generators producing and modulating basic bipedal locomotor pattern (Takakusaki et al., 2008). Movement initiation and anticipatory adjustments of ongoing motion in response to changing environment are achieved by cortical gait control. Descending tracts from distributed cortical areas, including supplementary motor area (SMA), primary motor cortex, and somatosensory cortex, project *via* the basal ganglia (BG) loop to adjust the activity of MLR/PPN by GABA-ergic inhibitory output of the substantia nigra pars reticulata (SNr; Sherman et al., 2015; Lewis and Shine, 2016; Weiss et al., 2020).

Evidence accumulates that the underlying pathophysiology of freezing arises from a complex disbalance within a distributed locomotor network (Nieuwboer and Giladi, 2013; Weiss et al., 2020). Recently, it has been proposed that a “circuitopathy” of the supra-spinal locomotor network including the sensorimotor cortex, BG, and midbrain locomotor centers is a key feature in the common neural pathway of FoG in PD (Lewis and Shine, 2016; Pozzi et al., 2019). Dopaminergic depletion of substantia nigra pars compacta leads to an overinhibitory activity of the SNr resulting in an excessive suppression of the MLR (Sherman et al., 2015). In parallel, overactive nigral output strongly inhibits thalamocortical projections which interrupt cortical gait control (Snijders et al., 2016).

Several neurophysiological studies have highlighted the role of over-synchronized oscillatory activity within the cortico-BG network in the pathophysiology of motor and non-motor impairments in PD (see review Oswal et al., 2013). Recently, Pozzi et al. (2019) revealed a sudden and transient breakdown in functional connectivity between motor cortex and STN for the time of motor block in PD patients in the theta-alpha band within the more affected hemisphere which was already present during the transition from walking to FoG. Of interest, directionality analysis revealed that the pathologically increased synchronization within the cortico-subcortical network during resting-state is mostly driven by abnormal cortical activity (Litvak et al., 2011; Sharott et al., 2018; Cagnan et al., 2019) further emphasizing the important role of sensorimotor cortex failure in the underlying mechanism of freezing.

Deep brain stimulation of the STN (STN DBS) may improve certain spatial aspect of parkinsonian gait disturbance especially in early years after DBS implantation but usually fails to modulate temporal gait characteristics (Pötter-Nerger and Volkmann, 2013). In particular, STN DBS reduces dopamine-responsive OFF freezing (Fasano et al., 2012) but has limited therapeutic effect on dopamine refractory FoG. Clinical response to STN DBS has shown to be correlated to its modulatory effects on functional connectivity within the sensorimotor cortex (Weiss et al., 2015).

In view of apparently untreatable gait impairments under STN DBS, multi-site DBS such as the co-stimulation of the STN and of the substantia nigra (STN+SN DBS) moved into focus. From a pathophysiological perspective, additional SNr stimulation is supposed to suppress pathological nigral activity and thereby may reduce excessive inhibition of the brainstem locomotor centers through overactive GABA-ergic SNr-PPN-projections (Snijders et al., 2016). In clinical practice, simultaneous high-frequency STN and SN stimulations are realized by co-activating the most caudal contact of the STN DBS electrode when located in the SN area. Recent intraoperative microelectrode recordings of the SNr during test stimulation seem to confirm the DBS-induced neural inactivation of SNr neurons (Milosevic et al., 2018). Nevertheless, the modulatory effect of STN+SN DBS on cortical-subcortical network level has not been investigated yet. So far, there are few case series (Weiss et al., 2011; Brosius et al., 2015) and one double-blinded cross-over study on STN+SN DBS (Weiss et al., 2013) suggesting an improvement of FoG by additional nigral stimulation. Currently, randomized multi-center data of STN+SN DBS are being analyzed (clinical trial registration number NCT02588144). Of interest, high-frequency SN stimulation showed the beneficial effect on bilateral temporal gait coordination (Scholten et al., 2017) which plays an important role in the temporal evolution of FoG (Plotnik et al., 2005; Chee et al., 2009).

Simultaneous EEG and kinematic measurements in PD patients during active DBS offer the opportunity to investigate the cortico-subcortical network by measuring activity changes in the cortical locomotor network in response to DBS-induced modulation of important subcortical locomotor hubs as the STN and the SN. The goal of this study was first to analyze the effect of STN+SN DBS compared to STN DBS on temporal gait characteristics in PD patients and second, to characterize cortical activity changes induced by additional SN stimulation during resting-state, effective lower limb stepping, and freezing episodes. We hypothesized that additional high-frequency stimulation of the SN leads to a disinhibition of both mesencephalic locomotor area and thalamo-cortical projections. This DBS-induced modulation of the cortical-BG-mesencephalic locomotor network might result in an improvement of gait function and might be quantifiable by changes in oscillatory activity over cortical areas.

## Material and Methods

This study was conducted in agreement with the Code of Ethics of the World Medical Association (World Medical Association Declaration of Helsinki, 2013) and was approved by the local ethics committee (reference: PV5281). All participants gave their written informed consent before taking part in the study.

### Subjects

A total of 12 patients with idiopathic PD (11 male, age: 66.5 ± 7.6 years, disease duration: 14.8 ± 4.7 years, Montreal Cognitive Assessment (MoCA) score: 26.9 ± 1.9) with chronically-implanted DBS electrodes, (time with DBS: 4.0 ± 3.8 years), were assessed and compared to 12 age-matched healthy controls (all male, age: 61.3 ± 7.5 years, MoCA score: 27.3 ± 1.4). PD patients were considered suitable candidates for the experimental protocol when the conditions were met that: (i) bilateral STN DBS electrodes were implanted for at least 6 months, and their lowermost contacts were localized within the dorsal substantia nigra; (ii) patients were able to stand and walk for at least 1 min without external assistance; (iii) did not present any competing neurological diseases or other gait-affecting musculoskeletal impairments by the time of testing; and (iv) the dopaminergic medication remained unchanged in the preceding 4 weeks. Preoperatively, all PD patients were screened and selected for DBS surgery in accordance with the common guidelines of DBS surgery (Defer et al., 1999). All patients were tested on their regular dopaminergic medication. As the PD patients selected for the study suffered from levodopa-resistant FoG, we expected no relevant medication-induced changes in stepping performance or cortical activity during a motor task. Table 1 provides further details of the clinical characteristics.

**Table 1:** Clinical and demographic characteristics of PD patients and healthy matched control persons.

	**PD patients**			**Healthy controls** (*n* = 12)
	**All** (*n* = 12)	**Freezers (*n* = 7)**	**Non-freezers (*n* = 5)**
Age [years]	66.5 ± 7.6	68.6 ± 3.1	63.6 ± 3.0	61.3 ± 7.5
MoCA	26.9 ± 1.9	26.6 ± 0.8	27.4 ± 0.7	27.3 ± 1.4
Disease duration [years]	14.8 ± 4.7	15.7 ± 2.1	13.6 ± 3.4	*n.a.*
Postoperative time [months]	48.2 ± 46.0	48.6 ± 22.4	47.6 ± 23.6	*n.a.*
Hoehn and Yahr	2.4 ± 0.8	2.7 ± 0.4	2.00 ± 0.0	*n.a.*
MDS-UPDRS III
STIM OFF	42.9 ± 10.9	45.1 ± 12.9	39.8 ± 9.4	*n.a.*
STN DBS	28.5 ± 10.5	31.7 ± 12.5	24.0 ± 7.4
STN+SN DBS	29.3 ± 11.7	33.0 ± 13.1	24.0 ± 9.5
Stepping variability
STIM OFF	14.2 ± 7.4	17.7 ± 7.9	9.2 ± 1.7	8.1 ± 1.8
STN DBS	14.5 ± 6.8	17.4 ± 5.8	10.4 ± 6.4
STN+SN DBS	13.0 ± 6.3	16.8 ± 5.6	7.7 ± 1.2
Stepping asymmetry
STIM OFF	14.1 ± 13.5	21.2 ± 13.6	4.1 ± 3.6	7.9 ± 5.1
STN DBS	12.0 ± 9.7	15.5 ± 8.3	7.0 ± 10.0
STN+SN DBS	14.0 ± 11.6	19.1 ± 7.9	7.8 ± 5.1
Mean FoG duration [s]
STIM OFF	*n.a.*	4.1 ± 3.4	*n.a.*	*n.a.*
STN DBS		2.2 ± 2.6
STN+SN DBS		6.8 ± 8.4
Number of FoG episodes
STIM OFF	*n.a.*	1.9 ± 1.7	*n.a.*	*n.a.*
STN DBS		1.0 ± 1.8
STN+SN DBS		1.6 ± 1.8

*In “Disease duration (years)”, the disease duration is calculated from the date of diagnosis to the date of baseline measurement. Abbreviations: FoG, Freezing of gait; MoCA, Montreal Cognitive Assessment score; MDS-UPDRS-III, motor-subscore (part III) of the Unified Parkinson’s Disease Rating Scale of the Movement Disorder Society; n.a., not applicable*.

### Surgery and Electrode Localization

All DBS systems were implanted at the Department of Neurosurgery at the University Medical Center Hamburg-Eppendorf, Germany. A detailed description of the surgical and lead placement procedure has been reported elsewhere (Sharott et al., 2014; Hidding et al., 2017; Dietrich et al., 2020). In brief, individual target coordinates for the dorsal STN were determined using preoperative magnetic resonance images fused with stereotactic computed tomography scans. In all patients, final electrode placement was adjusted according to intraoperative microelectrode recordings. The subthalamic and nigral region was mapped with sharp tungsten electrodes (Alpha Omega Inc., Nazareth, Israel) in up to five parallel tracks. Neurons of the sensorimotor STN were identified by tonic irregular oscillatory bursting activity in the range between 10 and 30 Hz and cell response to active and passive limb movements. A clear decrease of background noise and the emerging of high-frequency regular spiking activity signaled the entrance of the micro-tips into the SNr. Postoperatively, the reconstruction of the active DBS lead contacts (model 3389; Medtronic^®^, Minneapolis, MN, USA; electrode model 3389, Medtronic^®^, Minneapoli, MN, USA, in 10 cases, and electrode model 2202 and model 2201, Boston Scientific^®^, Valencia, CA, USA, in two cases) was performed by co-registration of the preoperative T1 MRI scans and post-operative CT scans using iPlan (iPlan stereotaxy; Brainlab, Feldkirchen, Germany). Further details concerning the localization of active electrode contacts are reported elsewhere (Hamel et al., 2003; Hidding et al., 2017, 2019).

### Experimental Protocol

Patients were evaluated in three DBS stimulation conditions in a pseudorandomized order with at least 45 min waiting period between conditions to prevent potential carry-over effects of the previous DBS setting: (i) with DBS switched off (STIM OFF); (ii) with DBS switched on with omnidirectional activation of the standard therapeutic contact located in the STN (STN DBS); and (iii) with DBS switched on with omnidirectional activation of two contacts for each lead: the standard therapeutic contact located in the STN, and one supplemental contact putatively located in the SN (STN+SN DBS). STN DBS and STN+SN DBS were standardized with a pulse width of 60 μs and a pulse frequency of 125 Hz and 130 Hz for Medtronic^®^ and Boston Scientific^®^, respectively. Active contacts and amplitudes were kept unchanged (i.e., the everyday therapeutic stimulation settings) to ensure the best individual STN stimulation. The combined STN+SN stimulation was provided by an additional activation of the lowermost contact using the “interleaving pulse” mode as suggested previously (Weiss et al., 2013) while active contacts and amplitudes of STN stimulation were held constant. Amplitudes for the “nigral” contacts were set according to a threshold testing for side effects prior to this study (Hidding et al., 2017). All stimulation settings were applied bilaterally. Details of DBS settings are provided in Table 2. We chose high-frequency DBS in both nuclei since this setting was most often used for the combined STN-SN stimulation mode in preceding studies. Patients and investigators were blinded to the counterbalanced sequence of stimulation conditions. To evaluate the clinical effectiveness of the different DBS stimulation settings, the motor sub-score of the Unified Parkinson’s Disease Rating Scale (MDS-UPDRS-III) was assessed by the same experienced investigator throughout the entire study. First, a 1 min seated resting-state task with eyes open was performed. Second, a Stepping in Place (SIP) task was used to simulate gait and provoke freezing episodes. SIP was used as it shared the same basic features of gait such as rhythmic alternating bilateral leg movements but could be performed under the restricted space condition during EEG recordings. Previous studies have demonstrated that forward walking and SIP showed similar altered temporal gait characteristics in PD (Syrkin-Nikolau et al., 2017) and that SIP effectively elicited FoG in PD freezers (Nantel et al., 2011; Fraix et al., 2013; Chomiak et al., 2015) which strongly correlated with patients’ self-report of freezing (Nantel et al., 2011). SIP was performed during: (1) continuous, regular stepping; and (2) stepping requiring sudden gait adjustments to increase susceptibility to FoG. More specifically, in task 1 the participants were instructed to execute alternating stepping movements in the upright position with their left and right leg at a self-paced, comfortable speed for 35 s. In task 2, stepping movements had to be adjusted in response to 10 auditory “start” and “stop” signals at various, randomized latencies with SIP intervals of 3–10 s. For each DBS condition, recordings lasted for about 5 min.

**Table 2:** Stimulation parameters for STN and STN+SN DBS.

		STN stimulation				Additional SN stimulation				Common settings			X/Y/Z coordinates
		Left electrode		Right electrode		Left electrode		Right electrode		Freq *[Hz]*	Pulse width *[μs]*		[mm]
ID	DBS syst	*Contacts*	*Amplitude*	*Contacts*	*Amplitude*	*Contacts*	*Amplitude*	*Contacts*	*Amplitude*		*left*	*right*
PD01	ME	2	3.2 V	10	3.0 V	0	1.5 V	8	1.7 V	125	60	60	L: 11.2 / 1.9 / 5.6 R: 8.3 / 5.5 / 4.0
PD02	ME	2	3.7 V	9	2.8 V	0	2.0 V	8	2.0 V	125	60	60	L: 10.9 / 2.2 / 4.7 R: 1.05 / 3.8 / 4.7
PD03	ME	1	3.3 V	9	3.1 V	0	1.5 V	8	1.5 V	125	60	60	L: 10.9 / 1.4 / 7.7 R: 11.1 / 2.7 / 6.7
PD04	ME	2	3.2 V	10	1.5 V	0	1.5 V	8	1.5 V	125	60	60	L: 11.9 / 2.2 / 5.2 R: 10.2 / 4.0 / 5.6
PD05	ME	2	1.6 V	10	2.4 V	0	1.0 V	8	1.0 V	125	60	60	L. 11.2 / 2.7 / 6.7 R: 8.2 / 1.6 / 4.4
PD06	ME	1	3.5 V	9	3.3 V	0	1.0 V	8	1.0 V	125	60	60	L: 11.3 / 2.2 / 6.2 R: 12.2 / 0.2 / 5.2
PD07	BS	5 / 6 / 7	2.7 mA	13 / 14 / 15	3.2 mA	1	3.5 mA	9	3.7 mA	130	60	60	L: 10.9 / 2.5 / 5.7 R: 10.4 / 0.4 / 5.2
PD08	BS	2 / 3	3.2 mA	10 / 11	4.0 mA	1	4.2 mA	9	5.0 mA	130	60	60	L: 8.81 / 3.4 / 7.4 R: 7.04 / 4.3 / 6.4
PD09	ME	1	1.7 V	10	3.0 V	0	1.0 V	8	1.0 V	125	60	60	L: 11.0 / 2.5 / 5.9 R: 10.1 / 1.3 / 5.0
PD10	ME	3	1.6 V	11	2.9 V	0	1.0 V	8	1.0 V	125	60	60	L: 9.5 / 2.8 / 6.4 R: 11.2 / 1.4 / 7.2
PD11	ME	3	3.5 V	10	3.3 V	0	0.7 V	8	0.7 V	125	60	60	L: 9.2 / 2.8 / 7.7 R: 10.2 / 2.4 / 6.0
PD12	ME	1	2.4 V	9	2.3 V	0	1.0 V	8	1.0 V	125	60	60	L: 10.7 / 5.3 / 6.9 R: 7.7 / 3.1 / 6.8

*STN+SN DBS was realized by additional activation of lowermost electrode contacts (Medtronic: contacts 0 and 8, Boston Scientific: contacts 1 and 9), while all other stimulation parameters used in STN DBS were held unchanged. Values reported are active contacts, amplitude (V = volts or mA = milliampere), pulse width (μs = microseconds) and stimulation frequency (Hz), for the left and right electrode. The neurostimulator case was always set as positive (anode) and the active contacts as negative (cathodes, contact number). For the left Medtronic (ME) electrode, contact 0 was the most ventral and contact 3 was the most dorsal. For the right ME electrode, contact 8 was the most ventral and contact 11 was the most dorsal. For the left Boston Scientific (BS) electrode, contact 1 was the most ventral and contact 8 was the most dorsal. For the right BS electrode, contact 9 was the most ventral and contact 16 was the most dorsal. Electrode coordinates are given in relation to the AC-PC line (mm) lateral to the midline (X), posterior to the mid-commissural point (Y) and inferior to the inter-commissural plane (Z)*.

### Data Acquisition

Stepping kinematics of lower limbs were recorded using two tri-axial accelerometers (MMA7260QT, Freescale Semiconductor Inc., Tempe, AZ, USA) attached to the outer foot ankles. Cortical activity was recorded using a 64-channel EEG system, with active ring electrodes mounted in accordance with the 10–10 system and referenced to the nose tip, including two additional EOG electrodes (EASYCAP GmbH, Herrsching, Germany). The electrodes had integrated impedance converters fitted directly into the electrode in order to minimize noise from the surrounding area as well as from movement artifacts. EEG and accelerometer signals were simultaneously recorded using BrainAmp amplifiers with analog bandpass filters set at 0.016–250 Hz and at 0.016–1,000 Hz, respectively, and a sampling rate of 2,500 Hz (BrainProducts, Munich, Germany). The frequency cutoff value of the high-pass was chosen to reduce artifacts from cable movements and channel drifts while minimizing data distortion. Cutoff frequencies for low-pass filtering were set to 250 Hz and 1,000 Hz for EEG and accelerometers respectively to ensure the retaining of the full range of physiological frequency spectra for further offline analysis.

### Behavioral Analysis

**Figure 1:**
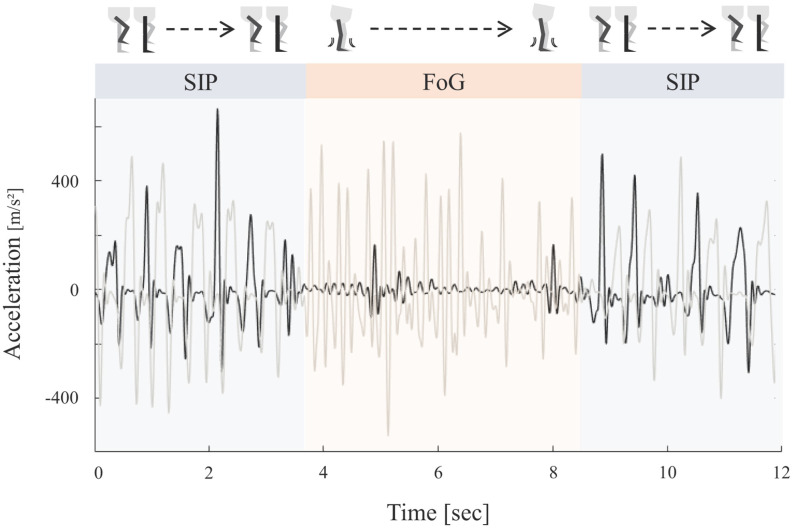
Schematic representation of the stepping in place (SIP) task and corresponding accelerometer curves. Example of vertical accelerometer signals of both legs (black = right leg, gray = left leg) during continuous SIP interrupted by a freezing episode. Effective stepping was characterized by alternating heel strikes. Freezing episodes were defined by high frequency low amplitude oscillations reflecting leg trembling during freezing of gait (FoG).

Heel strikes during SIP were automatically detected by a customized MATLAB script and checked by visual inspection. FoG episodes were identified based on the characteristic shift of frequency spectra of vertical leg acceleration towards higher frequency components compared to effective stepping (Figure 1; Moore et al., 2008). To this end, vertical accelerometer axes were bandpass filtered from 0.5 to 8 Hz and resampled at 100 Hz. Time-frequency transformation between 0.5 and 8 Hz was calculated using a Hanning-taper with a fixed time window of 4 s resulting in a frequency resolution of 0.5 Hz. For each point in time (10 ms), a freezing index (FI) was computed as the ratio between the square of the area under the power spectra in the “freezing” band (3.5 to 8 Hz) and the “locomotor” band (0.5–3 Hz). An individual freezing threshold was defined as a continuous period of time (≥3 s) in which FI was greater than the mean + 1 SD of the peak FI during standing before stepping initiation (Pozzi et al., 2019). This procedure has shown to be a reliable marker for objective freezing detection (Morris et al., 2012). All selected freezing episodes were verified by visual inspection of the data. Patients were labeled as “freezers” if they experienced freezing episodes in at least one DBS stimulation condition and labeled as “non-freezers” if they did not show FoG in any DBS setting. Effective stepping was defined as consecutive alternating heel strikes outside of FoG episodes. For each patient, the longest period of uninterrupted effective SIP was visually determined during the first block of 35 s of continuous SIP excluding the first 2 s after stepping initiation. Based on these predefined stepping episodes, temporal stepping parameters were explored. We focused on effects on stepping variability and symmetry, as FoG severity seems to correlate with gait asymmetry (Plotnik et al., 2005), and a previous study suggested a selective effect of nigral stimulation on temporal bilateral gait coordination (Scholten et al., 2017). First, subject’s mean step-to-step time for each foot was extracted from effective SIP episodes. Then, stepping variability (*var*) was analyzed as the *z*-transformed step-to-step time (*SPT*) coefficient of variability as


var=SD (SPT)/mean (SPT)×100

where higher value is associated with a higher degree of stepping variability. Stepping asymmetry (*asym*) was calculated as suggested in Plotnik et al. (2005).


asym=|ln (SSDT/LSDT)|

where *SSPT* and *LSPT* correspond to the leg with the shorter and longer mean stride time, respectively. An asymmetry index closer to 0 represents a more symmetric gait.

### Electrophysiological Analysis

EEG signals were pre-processed using the open-source EEGLAB toolbox (Version, 2020.0; Delorme and Makeig, 2004). To eliminate high frequency DBS artifacts, EEG data were low-pass filtered using a zero-phase Kaiser-windowed FIR filter at 100 Hz (pass-band 0–90 Hz, transition width 10 Hz, attenuation −60 dB). After down-sampling to 1,000 Hz, high-pass filtering at 0.75 Hz (zero-phase Kaiser-windowed FIR filter, stop-band 0–0.5 Hz, transition width 0.5 Hz, attenuation −60 dB) was performed to minimize slow drifts. Flat, noisy or uncorrelated channels were identified using the “clean_rawdata” EEGLAB plug-in, and replaced using spherical spline interpolation. On average, 58 out of 64 EEG channels per subject remained for further analysis (STIM OFF: 57 ± 4, STN DBS 58 ± 2, STN + SN DBS 58 ± 2). Artifact Subspace Reconstruction (ASR) was used to detect short-lasting artifacts in the data that most probably originated from muscular activity (Mullen et al., 2015). This principal component analysis (PCA) based method rejected non-stationary high-amplitude components and reconstructed channel data within a 0.5 s sliding window from remaining components. Rejection criterion was set to 15 SD of the mean amplitude of a clean portion of the same data. This threshold was in line with the one proposed in the literature (Chang et al., 2018) and visual inspection of the data before and after ASR ensured that clean portions of data were fully retained. EEG data were then re-referenced to a common average reference. Next, independent component analysis (ICA) was applied to decompose EEG signals. We used the adaptive mixture ICA method (AMICA) introduced by Palmer et al. (2008) which calculates temporal and spatial characteristics of independent components using a flexible sum of extended Gaussian models. AMICA was shown to outpower other common ICA methods in terms of component separation and dipole fitting (Delorme et al., 2012) and was successfully applied in a series of EEG analyses investigating human walking (Wagner et al., 2012, 2016, 2019). The number of independent components were reduced to the number of remaining eigenvalues of EEG data. Stereotypical artifacts including eye movements, blinks, ECG, DBS and muscular artifacts were removed based on their temporal characteristic and scalp projection. On average, 11 components per subject were rejected (STIM OFF: 11 ± 5, STN DBS 11 ± 5, STN+SN DBS 11 ± 6). Finally, a surface Laplacian transformation using the spherical spline method (lambda = 10^−5^, spline order = 4, spline iterations = 50, Perrin et al., 1989) was applied to further reduce movement artifacts and volume conduction. Cortical activity was analyzed using power-frequency spectra of absolute power. To compare episodes of effective SIP with different length among participants, EEG data were segmented in consistent non-overlapping epochs of 1 s length on subject level. For each epoch, power spectra were calculated using the open-source Fieldtrip toolbox (Version 20170607, Oostenveld et al., 2011). The embedded multitapers technique was used to increase statistic sensitivity and to compensate for small trial numbers. Spectral power analyses were performed between 4 and 45 Hz with a frequency resolution of 1 Hz and a frequency smoothing of ±2 Hz resulting in three Slepian sequence tapers being used. The absolute power spectra were transformed for normalization using natural logarithm and averaged within main frequency bands: theta (4–8 Hz), alpha (9–13 Hz), low-beta (14–20 Hz), high-beta (21–30 Hz), and gamma (31–45 Hz).

### Statistical Analysis

Statistical analyses were performed in SPSS Statistics 26 (IBM Coorp., New York, USA) and using the statistical methods as implemented in the FieldTrip toolbox for MATLAB^®^ (Maris and Oostenveld, 2007). All residuals were checked for normal distribution using Shapiro-Wilk tests. Biographic characteristics of PD freezers and non-freezers were compared using independent samples Mann-Whitney-U tests. To evaluate changes in UPDRS-III symptom scores between DBS settings a non-parametric Friedman test was conducted followed by Wilcoxon sign-rank tests for *post hoc* pairwise comparison.

Mean freezing duration and number of freezing episodes were compared between DBS conditions using repeated-measures ANOVAs with the three-level within-subject factor “stimulation”. To analyze the effect of stimulation setting on each temporal gait parameter in comparison to healthy controls, three separate independent samples *t*-tests (STIM OFF, STN DBS, and STN + SN DBS vs. controls) were performed. To compare the effect of the DBS conditions on gait parameters, repeated-measures ANOVAs were performed with the 3-level within-subject factor “stimulation” (STIM OFF, STN DBS, STN+SN DBS) and the between-subject factor “freezing” (“freezers” vs. “non-freezers”). Planned *post-hoc* paired samples *t*-tests were used for pairwise comparisons between DBS settings.

We tested the effect of the three DBS conditions on cortical oscillatory activity with respect to the different motor states in PD patients. We analyzed patients’ spectral power distributions in resting-state and continuous SIP (STIM OFF, STN DBS, and STN+SN DBS) in comparison to healthy controls (HCs) and between each other. We then compared relative power changes between SIP and resting-state conditions separately for healthy controls and each DBS condition. To do so, we used the non-parametric cluster-based permutation statistics as provided by the FieldTrip toolbox (Maris and Oostenveld, 2007). This approach was chosen due to the exploratory nature of this study, as it ensured a comprehensive analysis of the data without *a priori* assumptions and at the same time controlled for multiple comparisons. For each frequency band and channel, a distribution of the chosen test statistic was built. Two or more neighboring channels falling below a *p*-value of 0.05 were clustered on basis of spatial and spectral similarities. The sum of test statistic values within each cluster was then computed. To correct for multiple comparisons the maximum cluster-level test statistic was calculated using 2,000 random permutations across participants. Clusters below an alpha level of α = 0.025 (each side) were considered significant. For comparison of cortical activity between HCs and PD patients during resting-sate and SIP t-value distribution were built using independent samples *t*-tests. Analysis of power modulation between resting-state and SIP was conducted using paired samples *t*-tests separately for controls and DBS settings. To assess differences in cortical activity between STIM OFF, STN DBS, and STN+SN DBS cluster-based permutation tests were performed using the embedded dependent ANOVA F-statistics with the within-subject factor “stimulation” (STIM OFF vs. STN DBS vs. STN+SN DBS).

To compare stimulation effects on cortical power during freezing episodes between STIM OFF, STN DBS, and STN+SN DBS power spectra were analyzed by conducting Linear Mixed Effects Models (LMMs) in order to compensate for different numbers of freezers in each DBS setting. To this end, we first calculated mean averaged power spectra at a central-midline region of EEG. This ROI included the averaged signal of “Cz” electrode and its six neighboring electrodes corresponding to “FCz” and approximately to the “C1”, “C2”, “CP1”, “CP2”, “CPz” position of the international 10–10 system (see pictogram in panel A of Figure 3). The central ROI was strategically positioned to sample activity from cortical areas with particular relevance to locomotion including the supplementary motor area, premotor cortex, and primary motor cortex (Nutt et al., 2011; Takakusaki, 2013, 2017; Snijders et al., 2016). All covariance structures for repeated measures and random effects embedded in SPSS were compared. We chose those covariance structures that provided the best model fit using likelihood ratio tests. If the model fit of covariance structures did not differ significantly, we compared the goodness of fit between models using the Akaike information criterion (AIC) and selected the model with the best relative fit to data. Random slopes were tested for each model using the same procedure. Restricted maximum likelihood was used for parameter estimation. Estimated marginal means were used for pairwise comparisons between DBS settings.

**Figure 2:**
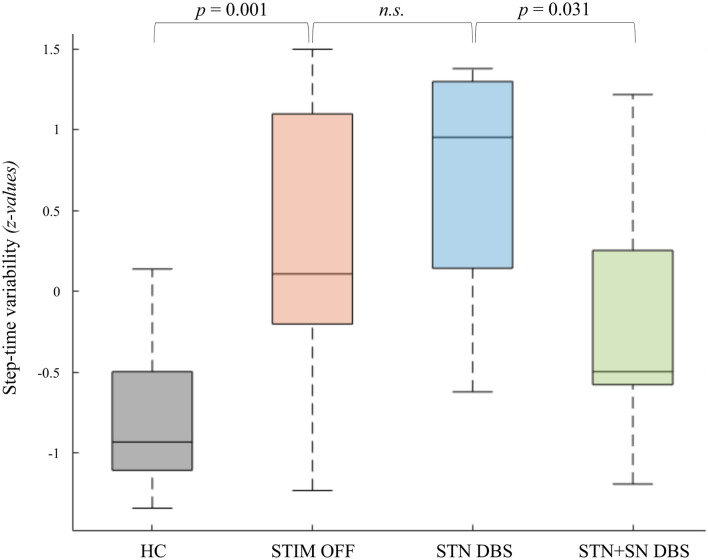
Deep brain stimulation (DBS) effects on step-time variability of the more affected leg. The coefficients of variability of step-to-step time in healthy controls and Parkinson’s disease (PD) patients in the three DBS conditions are displayed as boxplots. The values reported in the boxplots are median, interquartile range (IQR), whiskers (highest/lowest values of the data-set within 1.5 times of the IQR). Values reported above are significant *p*-values of planned *post-hoc* *t*-tests.

**Figure 3:**
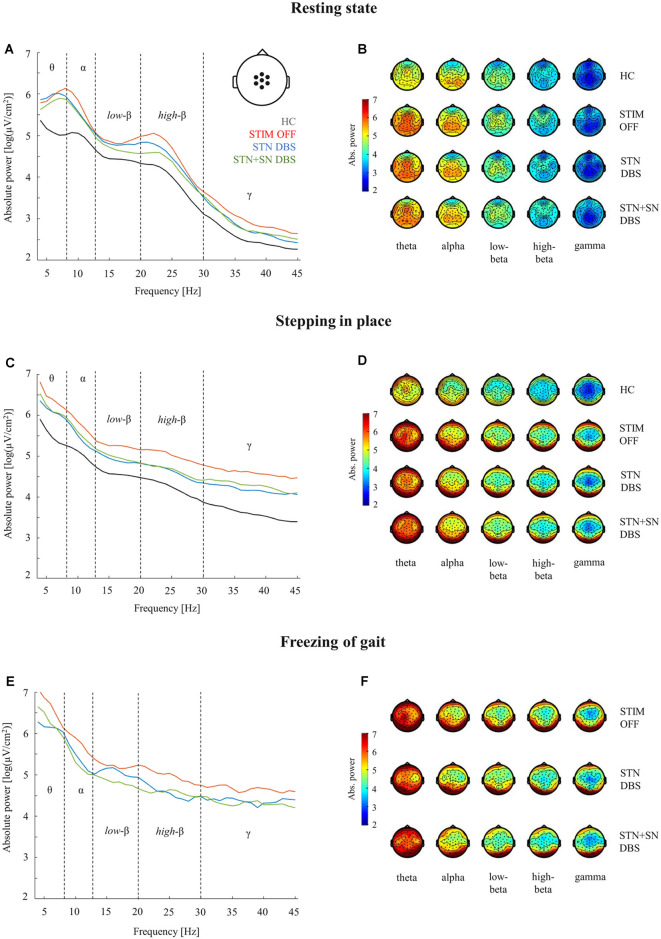
Cortical activity during resting-state, stepping in place, and during freezing of gait episodes. Analyses of absolute power in healthy control persons and PD patients for the three DBS conditions were performed in five frequency bands (theta 4–8 Hz, alpha 9–13 Hz, low-beta 14–20 Hz, high-beta 21–30 Hz, gamma 31–45 Hz). Panels **(A,C,E)**: comparison of absolute power spectra in the central ROI (pictogram in panel **A**). Panels **(B,D,F)**: topographies of absolute EEG power for healthy control persons **(B,D)** and for PD patients **(B,D,F)** during the three considered motor conditions.

## Results

### Clinical Characteristics and Freezing Severity

An overview of DBS effects on clinical and temporal gait characteristics is provided in Table 1. General motor performance as indexed by the MDS-UPDRS III was significantly improved by DBS (χ^2^(2) = 18.43, *p* < 0.001). STN DBS reduced the MDS-UPDRS III score by >30% compared to the baseline condition with DBS switched off (STIM OFF vs. STN DBS *Z* = −3.07, *p* < 0.001) indicating sufficient therapeutic effect and correct DBS lead position within the STN. The STN+SN DBS condition also significantly improved MDS-UPDRS III scores compared to baseline (STIM OFF vs. STN+SN DBS *Z* = −3.07, *p* < 0.001). There was no significant difference in MDS-UPDRS III scores between STN DBS and STN+SN DBS (*Z* = −0.49, *p* = 0.327). Based on biomechanical analyses, seven PD patients were identified as freezers as they showed at least one freezing episode in one of the DBS conditions during SIP. Five PD patients were identified as non-freezers as they performed the SIP tasks without any freezing episode. PD freezers and non-freezers did not reveal any significant group differences in terms of age, disease duration, time with DBS, MoCA score or Hoehn and Yahr disease stage (all *p*-values > than 0.1). In summary, six PD patients experienced 13 FoG episodes during STIM OFF with a total FoG duration of 82.3 s, whereas during standard STN DBS only three of 12 patients showed freezing behavior with a total number of seven freezing episodes and with a cumulative freezing duration of 50.5 s length. During combined STN+SN DBS five patients experienced a total of 11 FoG episodes with a duration of 70.5 s. Due to high interindividual variance in therapeutic response there was no significant main effect for stimulation on both FoG frequency (*F*_(2,12)_ = 0.923, *p* = 0.424) and FoG duration (*F*_(2,10)_ = 0.964, *p* = 0.414).

### Temporal Gait Characteristics

We tested whether temporal gait characteristics were affected by different DBS settings. Comparing HCs and PD patients during STIM OFF condition, PD patients showed a significant lower step-to-step time (*t*_(22)_ = −4.27, *p* < 0.001) reflecting parkinsonian gait with small shuffling steps. Furthermore, PD patients were characterized by a temporal gait dysregulation with significant higher temporal variability during effective SIP (*t*_(22)_ = 2.66, *p* = 0.014) and a slightly, non-significant increase in stepping asymmetry (*t*_(22)_ = 1.94, *p* = 0.076) compared to HCs. Subgroup-analysis revealed that PD freezers in STIM OFF showed a significant higher degree of stepping variability (*t*_(22)_ = 2.66, *p* = 0.014) and stepping asymmetry (*t*_(7.02)_ = 2.50, *p* = 0.041) compared to HCs. In contrast, non-freezers and HCs did not differ significantly with regard to temporal stepping parameters. Repeated-measures ANOVA revealed a clear between-subject effect showing significantly higher step-to-step time variability in freezers compared to non-freezers during effective SIP, independent of DBS condition (*F*_(1,10)_ = 34.07, *p* < 0.001, η^2^ = 0.77) emphasizing temporal gait parameters as suitable surrogate markers for freezing severity. STN DBS failed to improve both stepping variability and stepping asymmetry with respect to STIM OFF. However, combined STN+SN DBS led to a reduction in stepping variability compared to standard therapeutic STN DBS, which was statistically significant for patients’ more affected leg (*t*_(11)_ = 2.47, *p* = 0.031) suggesting a modulatory effect of STN+SN DBS on temporal gait integration in PD patients (Figure 2).

### Cortical Activity in STIM OFF During Resting-State and Effective Stepping

**Figure 4:**
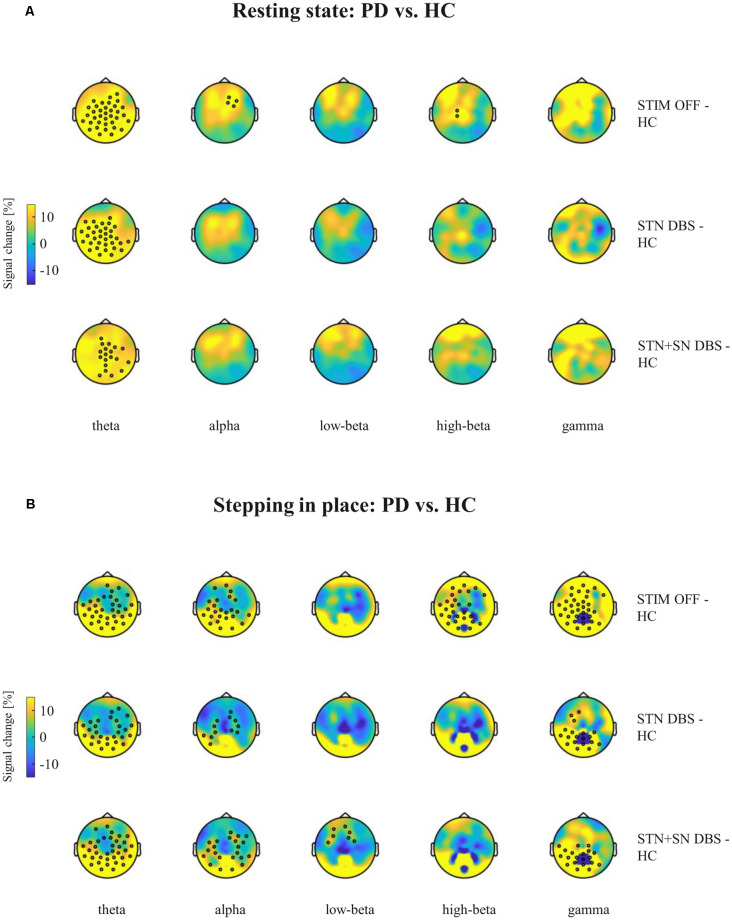
Cortical activity differences between PD patients and healthy controls (HCs) during resting-state and stepping in place. Topographic percentual power signal change between HCs and PD patients in the three DBS settings (rows) is displayed for **(A)** the resting-state condition and for **(B)** the stepping in place condition separately for the five frequency bands (columns). Black circles represent significant clusters of EEG sensors showing significant signal changes between motor conditions based on cluster-based permutation tests. In panel **(A)** the high-beta power cluster of midline-central channels was increased with *p*-value closed to the alpha level (*p* = 0.072).

**Figure 5:**
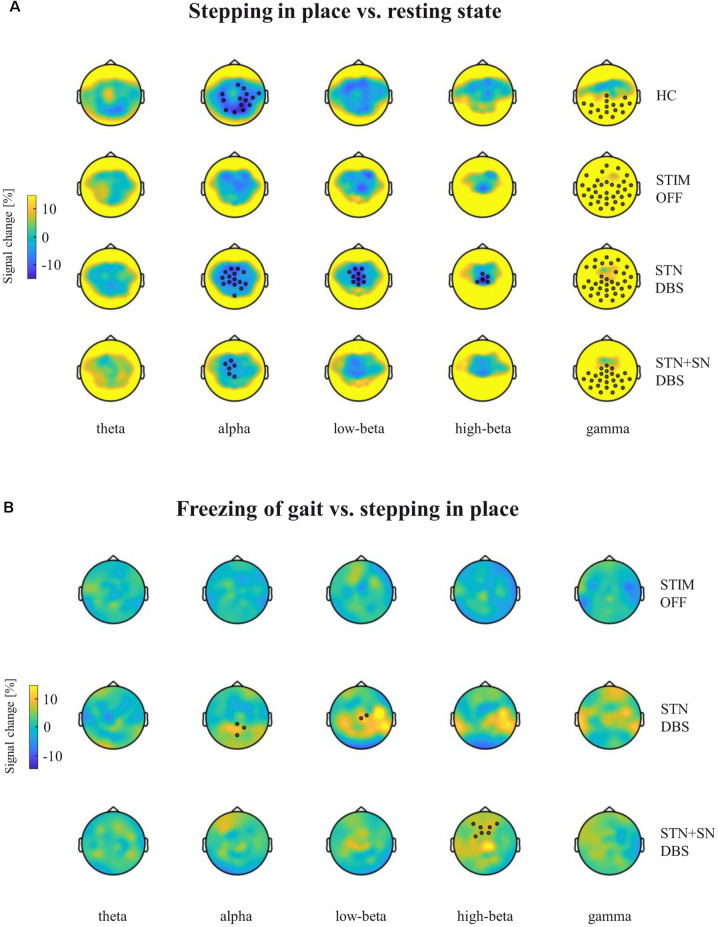
Cortical activity modulation in response to different motor conditions. Topographic percentual power signal change is displayed for **(A)** stepping in place vs. resting-state and for **(B)** freezing episodes vs. unaffected stepping in place. The comparisons are represented for healthy control persons and for PD patients in the STIM OFF, STN DBS, and STN+SN DBS conditions (rows) separated into five frequency bands (columns). Black circles represent significant clusters of EEG sensors showing significant signal changes between motor conditions based on cluster-based permutation tests.

High-density EEG recordings revealed that cortical oscillatory activity of PD patients and controls differed in the topographical distributions and absolute power levels, particularly at the central ROI between resting-state (Figures 3A,B), effective SIP (Figures 3C,D), and FoG episodes (Figures 3E,F). Cluster-based permutation test at rest revealed a significant power increase in low-frequency bands in untreated PD patients compared to HCs. In PD patients in STIM OFF, theta activity (*t* = 99.01, *p* = 0.008) at bilateral cluster of fronto-parietal electrodes and alpha activity (*t* = 11.98, *p* = 0.048) at right frontal cluster were significantly increased compared to controls. High-beta power in a cluster of midline-central channels was increased with *p*-value close to the alpha level (*t* = 8.64, *p* = 0.072; Figure 4A). During SIP, the cluster-based permutation tests still revealed elevated theta power at disseminated cortical clusters in PD patients with DBS switched off in comparison to control persons (STIM OFF vs. HC: *t* = 102.27, *p* < 0.001), elevated alpha power over bilateral frontoparietal sensors (*t* = 68.94, *p* = 0.006), increased high-beta power over left-lateralized clusters (*t* = 73.34, *p* = 0.003) and raised gamma activity (*t* = 57.80, *p* = 0.004) in PD patients in STIM OFF compared to controls (Figure 4B). Absolute theta power and beta power were not significantly modulated by continuous SIP compared to resting-state in PD patients in STIM OFF (Figure 5A). In HCs, alpha power was significantly desynchronized during SIP compared to resting over a right central-parietal cluster (*t* = −26.83, *p* = 0.011), but not in PD patients in STIM OFF condition. Gamma frequency power significantly increased during SIP compared to resting-state in both groups, HC and untreated PD, over a wide-spreading cluster of frontoparietal electrodes (HC: *t* = 58.48, *p* = 0.002, STIM OFF: *t* = 190, 48, *p* < 0.001; Figure 5A).

Summarizing, compared to healthy controls PD patients in the STIM OFF condition revealed increased, cortical absolute power in theta, alpha, and high beta frequencies in resting-state and effective stepping, and a reduced movement-related alpha desynchronization during SIP.

### Cortical Activity in Active DBS During Resting-State and Effective Stepping

In a second step, we focused on the effects of STN DBS and STN+SN DBS on cortical oscillatory power during resting-state and SIP. In the resting-state condition, cluster-based permutation tests revealed a significant increase in theta activity in PD patients in both active DBS conditions compared to healthy controls (STN DBS vs. HC: *t* = 91.26, *p* = 0.005; STN+SN DBS vs. HC: *t* = 62.14, *p* = 0.012). In contrast, resting-state alpha power and high-beta power were no longer increased in PD during STN DBS and STN+SN DBS conditions compared to controls indicating a modulatory effect on alpha and high-beta activity by both DBS settings (Figure 4A). In line with the results on resting-state, cortical activity during stepping in place was characterized by a significant and excessive increase in theta power independent of DBS settings over disseminated cortical clusters compared to HCs (STN DBS vs. HC: *t* = 88.20, *p* < 0.001; STN+SN DBS vs. HC: *t* = 134.53, *p* < 0.001; Figure 4B). Theta power was not significantly modulated during regular SIP compared to resting-state in PD patients with active DBS (Figure 5A). Alpha frequency power during SIP was still significantly increased in PD patients with active DBS compared to HCs (Figure 4B), this effect was most pronounced over bilateral central-parietal areas (STN DBS vs. HC, left cluster: *t* = 15.17, *p* = 0.031, right cluster *t* = 14.87, *p* = 0.032; STN+SN DBS vs. HC: *t* = 73.05, *p* = 0.003). However, alpha power was significantly desynchronized during SIP compared to resting-state in both active DBS conditions indicating a re-established movement-related cortical alpha modulation (STN DBS: *t* = −51.52, *p* = 0.004; STN+SN DBS: *t* = −11.95, *p* = 0.044; Figure 5A). During regular SIP, high-beta power was reduced toward the level of HCs during both, STN DBS and STN+SN DBS (Figure 4B). Low- and high-beta power at central sites were significantly desynchronized by SIP compared to resting-state in STN DBS condition (low-beta: *t* = −39.16, *p* = 0.004; high-beta: *t* = −18.56, *p* = 0.020; Figure 5A). Gamma frequency power significantly increased during SIP compared to resting-state in PD with STN DBS over a wide-spreading cluster of channels. STN+SN DBS was instead showing a more focal gamma power increase over a central-parietal cluster (STN DBS: *t* = 199.33, *p* < 0.001, STN+SN DBS: *t* = 132.04, *p* < 0.001; Figure 5A).

Summarizing the effect of DBS on cortical oscillatory power changes, we observed that both STN DBS and STN+SN DBS modulated cortical activity, resulting in a normalization of pathologically increased resting-state alpha and high-beta power. During SIP, both DBS settings successfully reduced exaggerated high-beta activity and restored the physiological, movement-related alpha desynchronization.

### Cortical Activity During Gait Freezing

In a third step, we assessed cortical activity changes during FoG episodes and the impact of the DBS conditions. We analyzed differences in cortical activity between regular SIP and FoG episodes within the PD “freezers” group using the cluster-based permutation test. In the STIM OFF condition, cortical activity did not differ significantly in any frequency band between regular SIP and FoG (OFF-FoG). However, FoG episodes that occurred during STN DBS (ON-FoG) were characterized by a significant increase in parietal alpha power (*t* = 13.56, *p* < 0.001) and central low-beta power (*t* = 19.24, *p* < 0.001) compared to regular SIP (Figure 5B). These effects were most obvious in midline-postcentral and midline-central areas, respectively. In contrast, ON-freezing during STN+SN DBS was characterized by a significant increase in high-beta power over a cluster of frontal sensors as during regular SIP (*t* = 11.64, *p* = 0.034; Figure 5B). To investigate differences in absolute cortical power between STIM OFF, STN DBS, and STN+SN DBS, we conducted LMMs for power differences over the predefined ROI located over central EEG signals that showed significant beta modulation during stepping in place in STN-DBS condition. For each frequency band, a random intercept fixed slope model with a scaled identity matrix as a repeated covariance type was used. Estimated marginal means of random intercept fixed slope LMM did not show significant group-level effects for any frequency band (all *p* > 0.1).

Summarizing, STN DBS, and STN+SN DBS induced spectrally and topographically different cortical activation patterns during FoG with re-emergence of parietal alpha and central low-beta activity with STN DBS and in contrast frontal high-beta activity during STN SN DBS.

## Discussion

In this study, we used a sensor-based analysis of a SIP task with simultaneous high-density EEG recordings in healthy controls and PD patients to evaluate the effects of conventional STN DBS and STN+SN DBS on temporal stepping characteristics and activity modulation of cortical nodes of the gait-network. At a behavioral level, STN+SN DBS was superior to STN DBS by modulation of temporal gait characteristics as a reduction of step time variability of the more affected leg. At the EEG level, we demonstrated that STN+SN DBS modulated cortical activity within the gait-network of PD patients. Both, STN DBS and STN+SN DBS normalized pathologically exaggerated alpha activity in PD patients compared to STIM OFF at rest. The excessive cortical high-beta activity was also successfully reduced to a similar extent by both active DBS setting. During FoG, STN DBS and STN+SN DBS revealed spectrally and topographically different cortical activity patterns with re-emergence of low-beta increase over the sensorimotor cortex with STN DBS and relative high-beta increase over frontal cortical areas with STN+SN DBS during motor blocks.

### Limitations and Methodological Considerations

There are certain limitations of the study. We used a gait-like movement, SIP, which is lacking the propulsive movement of real gait. We used SIP due to spatial constraints when recording simultaneously 64 channel EEG, but also since SIP was demonstrated to detect FoG in PD and since SIP correlated with subjectively perceived FoG severity as measured by FoG-Q (Nantel et al., 2011). SIP is therefore quite useful in the assessment of gait-like movements. Second, we assessed the PD patients with regular medication intake (MED ON), which might induce “ceiling effects” or residual fluctuations of the current motor state depending on the tablet wearing off. We chose the MED ON condition for three reasons. On the one hand, we aimed to assess the “real-world” condition, which might be easier to transfer into a clinical routine. On the other hand, we chose this MED ON condition to compensate for differences in symptom severity particularly FoG due to different disease severity leading to a more comparable baseline. Third, we were particularly interested in “ON-Freezing” which constitutes a particular clinical problem. Potential, residual medication-dependent fluctuations were clinically controlled and the DBS conditions were randomized to avoid order effects within the fluctuation state. Besides, the interpretation of EEG results needs to account for L-DOPA induced changes in cortical activity apart from DBS effects (Toledo et al., 2014).

### Behavioral Effects of DBS on Gait

Previous studies revealed heterogeneous effects of conventional STN DBS on gait. In the early stages of the disease, STN DBS can improve levodopa-responsive gait impairment (Pötter-Nerger and Volkmann, 2013). However, as the disease progresses, it is observed that axial symptoms become dopa-resistant and STN DBS might become less effective in terms of gait improvement (Krack et al., 2003; Rodriguez-Oroz et al., 2005; Schupbach et al., 2005). There are even reports of STN DBS induced FoG postoperatively (van Nuenen et al., 2008; Fleury et al., 2016). In objective gait analyses, levodopa-responsive parkinsonian gait impairments are improved by STN DBS with better spatial gait characteristics as stride length and velocity (Xie et al., 2001; Krystkowiak et al., 2003). However, temporal gait parameters such as step time, symmetry, and rhythmicity are not ameliorated by STN DBS (Faist et al., 2001). This is critical, as temporal gait dysregulation is one hallmark in the pathogenesis of FoG (Nutt et al., 2011). Of interest, a recent study suggested a beneficial effect of nigral neurostimulation on temporal gait regularization in PD (Scholten et al., 2017). There are different hypotheses on STN DBS-induced gait impairment. A recent study using normative connectomes based on MR images of PD patients after DBS implantation revealed deterioration of FoG postoperatively if the ansa lenticularis of capsula interna fibers were unintentionally stimulated by DBS (Strelow et al., 2021).

In the present study, we investigated the effect of subthalamic and nigral stimulation on temporal gait characteristics in a cohort of PD patients in advanced disease stages suffering from ON-medication/ON-stimulation gait freezing according to the self-evaluation provided with the FoG questionnaire. We demonstrated that combined STN+SN DBS significantly reduced step-time variability of the more affected leg compared to STN DBS alone in line with recent findings of nigral-specific effects on temporal gait characteristics (Scholten et al., 2017).

### What Are the Mechanisms of Action of STN+SN DBS?

One of the main goals of that study was to assess mechanisms of action of STN+SN DBS in the mediation of beneficial effects on gait and FoG. Originally, the new stimulation algorithm of STN+SN DBS was introduced in view of untreatable, residual gait symptoms and FoG under STN DBS (Weiss et al., 2013). The pathophysiological hypothesis for the use of STN+SN DBS in PD gait disorders was to intensify the release of the pathological inhibition of the BG-brainstem route. The SN is of particular interest since animal data suggest dense reciprocal interconnections between the SNr and mesencephalic locomotor region such as the PPN (Breit et al., 2001, 2005) projecting to spinal central pattern generators (Pahapill and Lozano, 2000). This subcortical BG route is assumed to be involved in automatic gait behavior including rhythmicity, posture preparations, and adjustments during locomotion (Takakusaki, 2013; Snijders et al., 2016; Marquez et al., 2020). However, automatic gait behavior is controlled by a multilevel network with cortical motor control centers involved in the initiation and adjustment of locomotion in environmental conditions as turning, stopping, or maneuvering obstacles (Marquez et al., 2020). The motor cortex is assumed to initiate gait by the descending command to release automatic rhythmic stepping patterns. The supplementary motor cortex is supposed to disconnect from BG resulting in the loss of internal cueing of automatic predefined motor stepping patterns and loss of automatic updating of motor programs (Marquez et al., 2020). In light of this fundamental role of the motor cortex in gait control, it was therefore of interest, whether STN+SN DBS mediates its beneficial gait effects not only by subcortical pathways but through cortical areas *via* BG thalamo-cortical projections.

### Absolute Power Increase in PD Patients in Comparison to HCs

An increase of absolute power across a broad range of frequencies was observed in PD patients, especially during the STIM OFF condition, when compared to HCs (Figures 3A,C). This general enhancement of absolute power in PD patients is in accordance with previous studies, which attributed this phenomenon to a pathophysiological chain reaction initiated by the effects of dopamine denervation in BG-thalamo-cortical loops (Tanaka et al., 2000; Moazami-Goudarzi et al., 2008; Gulberti et al., 2015). In particular, the slowing of EEG activity in comparison to HCs is a consistent finding in PD patients (Soikkeli et al., 1991; Stoffers et al., 2007), and an increased theta power has been associated with clinical measures of disease progression (Soikkeli et al., 1991; Bosboom et al., 2006; Stoffers et al., 2007; Serizawa et al., 2008) and cognitive decline (Neufeld et al., 1988, 1994; Tanaka et al., 2000; Sinanović et al., 2005; Olde Dubbelink et al., 2013; Guner et al., 2017). In the context of the so-called thalamo-cortical dysrhythmia framework, the pathological increase of low-frequency oscillatory bursting activity in thalamo-cortical circuits, leads to the pathological emergence of aberrant low- and high-frequency oscillations at the cortical level (Llinás et al., 2005; Moazami-Goudarzi et al., 2008). The DBS-induced inhibition of aberrant BG output to the thalamus may in turn reduce abnormal thalamo-cortical rhythmicity and normalize high beta oscillatory activities at the cortical level (Llinás et al., 2005), and thus it may also facilitate stepping movements (see Figures 3C,D and Figure 4B).

### Oscillatory Cortical Activity in PD Motor Control and Regular Stepping

To integrate the findings of specific cortical oscillatory power changes by STN+SN DBS compared to conventional STN DBS in the mediation of clinically beneficial effects, one needs to consider the current framework of spatially and spectrally segregated oscillatory activity changes within cortico-BG circuits in the pathophysiology of motor and non-motor parkinsonian symptoms (Oswal et al., 2013).

In PD at rest, excessive beta activity dominates within the cortico-subthalamic network involving motor and premotor areas (Hammond et al., 2007). There are further oscillatory activity changes in PD in other frequency bands within the subcortico-cortical loops as widespread alpha band (Litvak et al., 2011), gamma-band (Jenkinson et al., 2013), or theta band changes (Oswal et al., 2013). Within these subcortico-cortical networks, it was demonstrated, that cortical beta or lower band oscillations were the most likely driver or “master” and subthalamic oscillatory activity was the “slave” driven by the cortical control (Litvak et al., 2011). In contrast, gamma band activity was proposed to be driven “bottom-up” subcortically and driving higher cortical centers (Jenkinson et al., 2013).

In detail, the beta band activity is probably the best-investigated frequency band and is assumed to represent a hallmark of dopamine depletion in the bradykinetic parkinsonian pathophysiological state (Little et al., 2012). Spontaneous subthalamic fluctuations of beta activity were shown to correlate with the clinical state as bradykinesia (Kühn et al., 2009; Little et al., 2012). Non-invasive magneto-encephalography highlighted the exaggeration of beta band activity over motor cortical areas at rest in PD correlating with motor impairment (Stoffers et al., 2008; Pollok et al., 2012). Between cortical and subcortical sites, beta activity is pathologically synchronized within the BG loop in PD (Williams et al., 2002; Hirschmann et al., 2011; Litvak et al., 2011). Prior to self- and externally paced movements, oscillations in the beta band are suppressed in the subthalamic nucleus and globus pallidum (Levy et al., 2002; Kuhn et al., 2004), as well as in the sensorimotor cortex (Pfurtscheller, 1981; Crone et al., 1998; Oswal et al., 2013). It was therefore proposed that in PD, bradykinesia might be due to the inability of the BG to release the cortical information flow during movement (Brown and Marsden, 1998) resulting in a blockade of information transfer through BG-cortical projections.

In this experiment, we could transfer those findings from general motor control onto SIP as a specific, gait-like movement. At rest, we observed pathologically enhanced high-beta activity at central channels in PD patients compared to HCs associated with a deterioration of flexible movement initiation. With movement-onset during SIP, cortical beta activity was desynchronized in healthy controls, but not in PD patients in STIM OFF reflecting the inability to release new motor commands through cortical areas.

Since beta oscillations play a predominant role in the pathophysiology of PD, it is of interest whether subcortico-cortical beta oscillations are modulated by therapeutical DBS. The effect of conventional STN DBS on beta oscillations within the BG-cortical loop has been intensively assessed. STN DBS suppresses the pathologically increased beta oscillations in the STN (Kuhn et al., 2008; Eusebio et al., 2011), GPI (Brown et al., 2004) and reduces the coherence in the beta band between the motor cortex and the STN (Kuhn et al., 2008). Thus, STN DBS exerts its effects locally at the site of stimulation within the STN and over functionally connected elements of the cortex-BG network. These STN DBS induced changes in beta oscillations and beta coherence were negatively correlated with movement amplitude (Kuhn et al., 2008).

Here we assessed whether there is therapeutic modulation of beta power at the cortical sites with STN DBS and STN+SN DBS within a stepping movement. At rest and during regular SIP, the pathologically enhanced cortical beta activity was reduced during STN DBS and STN+SN DBS compared to STIM OFF indicating that both DBS modes improve the resolution of beta-associated communication blocks and efficient release of motor programs to the motor cortex during regular SIP. Still, STN DBS and STN+SN differed topographically in their control of beta activity indicating possible different channels in the mediation of beneficial effects.

Alpha activity has regained interest in the understanding of PD pathophysiology. On the one hand, there is a diffused increase of cortical background alpha activity and even slower frequency bands in PD (Stoffers et al., 2007; Stam, 2010). Alpha activity is particularly present in a network between the STN, temporo-parietal and brainstem areas (Hirschmann et al., 2011; Litvak et al., 2011). At rest, coherent alpha oscillations were observed in the STN and at various locations in the ipsilateral temporal lobe (Hirschmann et al., 2011). Alpha activity has been proposed to be involved in orienting attention at a cortical level (Klimesch, 2012). It was proposed that alpha-band oscillations are involved in suppression and selection of actions which are closely linked to the fundamental functions of attention to enable controlled knowledge access and provide time, space, and context orientation (Klimesch, 2012).

We found the alpha activity to be modulated by task-condition and DBS. In HCs and PD patients treated with STN DBS or STN+SN DBS, alpha oscillatory power was reduced during SIP compared to rest, still, alpha activity levels in patients were generally higher compared to controls. During STN DBS and STN+SN DBS, stepping-induced alpha power reduction was more intense compared to STIM OFF in central clusters. This might indicate enhanced attentional resources in both DBS conditions which might be beneficial in the maintenance of stepping quality and prevention of FoG (Yarnall et al., 2011; Tessitore et al., 2012).

Particular attention has been paid to gamma band activity as a key element of higher brain function, participating in arousal, perception, executive function, memory (Garcia-Rill et al., 2019), and vigor of the motor task (Joundi et al., 2012; Jenkinson et al., 2013). Gamma band activity is an inconsistent, broad-band feature at rest, but most obvious during voluntary movement (Androulidakis et al., 2007; Jenkinson et al., 2013), that is recordable as synchronized activity throughout different sites of the BG loop (Alegre et al., 2005) including cortical areas (Lalo et al., 2008; Litvak et al., 2012). There is evidence of coherent gamma activity between STN and mesial and lateral cortex with symmetrical bidirectional coupling after dopaminergic therapy (Lalo et al., 2008). Activity in the gamma range seems to be primarily physiological as it can be recorded in healthy animals (Berke, 2009) or humans without PD (Ball et al., 2008; Cheyne et al., 2008). In PD, gamma activity is decreased without medication (Mazzoni et al., 2007) and increased following levodopa administration (Brown et al., 2001; Alegre et al., 2005). The extent of gamma power increase correlated with motor improvement (Kuhn et al., 2006). The gamma band was therefore proposed to be a “pro-kinetic” oscillatory activity. Gamma band activity was proposed to be mediated by “bottom-up” brain processing to communicate sensory events to higher centers to promote perception and arousal (Garcia-Rill et al., 2019). One important brainstem nucleus involved in the mediation of gamma activity seems to be the PPN with its ascending, widespread projections through intralaminar, parafascicular, and center median thalamic nuclei (Scarnati et al., 1987; Capozzo et al., 2003) to the cerebral cortex and BG structures (Steriade and Glenn, 1982; Otake and Nakamura, 1998). We found an increase in gamma oscillatory power in HCs and PD patients during SIP compared to rest emphasizing the prokinetic feature of gamma band activity. These gamma band changes differed between STN DBS and STN+SN DBS conditions with a more widespread topographically pattern of gamma modulation during STN DBS and a more focal gamma increase over central and parietal clusters during STN+SN DBS underlining potential differences in mechanisms of action of these two stimulation modes. Since the PPN is assumed to play a major role within the mediation of gamma oscillatory activity to higher cortical centers and given the strong interconnections of PPN and SNr, one could assume that these direct SNr-PPN projections might be particularly modulated by STN+SN DBS resulting in specific cortical gamma oscillatory patterns.

### Cortical Oscillatory Activity Changes During FoG in PD

In light of cortical oscillatory activity changes during regular stepping and its modulation by DBS, it is of particular interest how the cortical activity is modulated during FoG. Recently, cortical alpha and beta band oscillatory power were assessed in more detail in freezing PD patients. Summarizing the different results, there were two main findings. On the one hand, there were significant increases in beta power associated with FoG compared to regular walking. On the other hand, there was an increase of lower frequency as alpha activity in FoG and even in the transition phase to FoG.

In one study, EEG signals during effective walking, FoG, and transition to FoG were analyzed with mobile EEG (Shine et al., 2014). There was an increase in cortical beta activity when comparing freezing to regular walking in the frontal lead or freezing to the transition phase in the parietal lead. This was interpreted as impaired or blocked communication of frontally generated motor plans to the motor cortex, leading to gait impairment (Shine et al., 2014).

This effect could be replicated in another study with two PD groups of freezers and non-freezers during a lower-limb pedaling task (Singh et al., 2019). Freezing PD patients exhibited increased beta-band (13–30 Hz) power at mid-frontal electrode Cz during pedaling compared to the non-freezing group. This increment of cortical beta was shown to be accompanied by increased subthalamic high-beta activity in PD patients with freezing in OFF dopaminergic medication compared to non-freezers (Toledo et al., 2014). This increased beta activity was shown to be modulated by therapeutic intervention. After the application of L-Dopa, the high-beta power at cortical and subcortical sites in freezers was reduced, which was accompanied by clinical improvement with FoG event cessation (Toledo et al., 2014).

In congruence with these previous findings, we found a re-emergence of beta oscillatory activity at cortical sites during FoG compared to regular SIP in all DBS conditions. FoG might therefore represent a “breakdown” of the subcortico-cortical loop by exaggerated beta oscillatory activity stopping the release of motor programs for gait initiation and gait performance. The hypothesis on pathologically increased beta activity resulting in the abnormal persistence of the status-quo of the movement state (Engel and Fries, 2010) fits FoG phenomenology with the persistence of continued, inveterate movement arrest of the lower limbs. Clinically, FoG episodes were more frequent during STIM OFF than during both DBS active conditions, still, we found the re-emergence of beta activity in all DBS conditions with the same behavioral output of motor arrest during FoG. We propose the hypothesis, that STN DBS and STN+SN DBS might act on gait control and regular stepping by promoting a “beta-resilience” or “beta-buffer” by restauration of the movement-related beta desynchronization. This DBS-induced “beta-buffer” needs to be depleted before a threshold is passed resulting in FoG.

Interestingly, we observed different patterns of beta activity re-emergence in the two different DBS conditions. During STN DBS there was a reappearance of low-beta activity in central clusters, whereas during STN+SN DBS there was re-emergence of high-beta activity frontally. The role of low-beta and high-beta activity is not yet completely understood, still, it was proposed that low-beta and high-beta oscillations carry independent information about movements as observed during reach-to-grasp tasks (Vissani et al., 2021). It was proposed that low-beta oscillations convey information about the principal movement state and high-beta activity is more informative of the details of the different active movement phases (Vissani et al., 2021). It might be hypothesized, that STN DBS and STN+SN DBS might act differentially on cortical activity through different beta frequency band “channels”.

Besides, previous studies on FoG emphasized changes in low frequency band activity. Alpha power and theta activity in the central and frontal leads were shown to be increased during FoG and even during the transition from normal walking to freezing (Shine et al., 2014). These findings of increased low frequency power, particularly theta activity, were interpreted as an increased cognitive load while conflict-processing directly before and during FoG since increment of low frequency oscillations has been associated with the performance of cognitive tasks (Basar et al., 2001), as the processing of conflict (Shine et al., 2013c) and cognitive interference (Lewis and Barker, 2009; Nigbur et al., 2011). This EEG finding of the relation of conflict-related signals indexed by increased low frequency changes in a network of fronto-parietal regions and FoG is in line with previous neuroimaging studies of FoG (Shine et al., 2013a, b). This finding could be also replicated for freezing of the upper limb in PD patients performing continuous tapping of the right index finger (Scholten et al., 2016). Freezing episodes of the upper limb were associated with increased cortical activity at 7–11 Hz. During the transition from regular tapping to “freezing” the cortical activity first increased over the left sensorimotor area followed by a spread to the left frontal and right parietal areas. In the STN DBS condition, we observed during FoG episodes a significant increase in alpha activity in the midline–parietal clusters. This finding is in line with the previous assumptions, that cortical conflict-processing reflected by cortical lower band activity enhances the risk for the occurrence of FoG and underlines the importance of cognitive-motor interference in the pathogenesis of FoG.

In summary, we found a superior behavioral effect of STN+SN DBS compared to conventional STN DBS on temporal SIP characteristics, which were accompanied by distinct cortical oscillatory patterns of low- and high-beta bands during SIP and FoG.

## Data Availability Statement

The raw data supporting the conclusions of this article will be made available by the authors, without undue reservation.

## Ethics Statement

The studies involving human participants were reviewed and approved by Ethikkommission der Ärztekammer Hamburg. The patients/participants provided their written informed consent to participate in this study.

## Author Contributions

The work presented here was carried out in collaboration between all authors. (1) Research project: A. Conception: JW, AG, and MP-N designed methods and assessments. B. Organization: JW, MS, WH, MW, CG, AE, CM, AG, and MP-N. C. Execution: JW, MS, WH, CM, AG, and MP-N. (2) Statistical analysis: A. Design: JW, AG, and MP-N. B. Execution: JW analyzed the data. C. Review and critique: JW, AG, and MP-N discussed analyses, interpretation and presentation. (3) Manuscript: A. Writing of the first draft: JW, AG, and MP-N. B. Review and critique: JW, MS, WH, MW, CG, AE, CM, AG, and MP-N. All authors contributed to the article and approved the submitted version.

## Conflict of Interest

The authors declare that the research was conducted in the absence of any commercial or financial relationships that could be construed as a potential conflict of interest. MW and AE declare no relevant conflicts of interest. Some of the authors (MS, WH, CM, and AG) have occasionally been reimbursed for travel expenses from Medtronic Inc. CG reports personal fees and other from Bayer Healthcare and Boehringer Ingelheim, personal fees from Abbott, Amgen, BMS, Sanofi Aventis, and Prediction Biosciences. CM received lecture, teaching and proctoring fees from Abbott. WH received lecture fees and honoraria for serving on advisory boards and travel grants from Boston Scientific, Medtronic, and Abbott. MP-N received lecture fees from Abbott and Licher, and served as consultant for Medtronic, Boston scientific, and Abbvie.

## Publisher’s Note

All claims expressed in this article are solely those of the authors and do not necessarily represent those of their affiliated organizations, or those of the publisher, the editors and the reviewers. Any product that may be evaluated in this article, or claim that may be made by its manufacturer, is not guaranteed or endorsed by the publisher.
